# Building a Historic Population Platform

**DOI:** 10.23889/ijpds.v9i5.2532

**Published:** 2024-09-18

**Authors:** Charini Nanayakkara, Peter Christen, Chris Dibben, Lee Williamson, Eilidh Garrett

**Affiliations:** 1 Australian National University; 2 University of Edinburgh

## Objectives

With the increasing digital availability of large population databases of historical census or vital event records, the tasks of storing, cleaning, processing, linking and analysing such data become more challenging. Suitable computing platforms and software systems are required to handle such databases, and facilitate the application of complex record linkage algorithms, for example to reconstruct populations that cover a full country over many decades. We present our efforts to achieve these goals on a database of over 20 million vital event records spanning over 120 years to create a “Historic Population Platform” (HiPP).

## Approach

We created a graph database using the Neo4J software, where each birth, death and marriage certificate is represented as a node. We then generated actor nodes from these certificates which represent individuals (such as birth babies and their parents, or marriage brides and grooms). Data cleaning steps included the correction and imputation of invalid, corrupted, and missing age and date values using information from related certificates.

## Results

Our initial graph database contains over 100 million nodes and nearly 200 million edges, while our data cleaning methods help to substantially increase the number of valid age and date values.

## Conclusion

The availability of large historical population databases provides exciting opportunities for social science and health research. However, existing methods have limitations in handling data quality and the sizes of such databases. We presented novel methods to deal with these challenges which we hope will be of use for other projects that aim to build a HiPP.

